# An assessment of measured and computed depth of closure around Japan

**DOI:** 10.1038/s41598-020-59718-5

**Published:** 2020-02-19

**Authors:** Keiko Udo, Roshanka Ranasinghe, Yuriko Takeda

**Affiliations:** 1 International Research Institute of Disaster Science, Tohoku University, 468-1 Aoba, Sendai, 980-8572 Japan; 2 Department of Water Science and Engineering, IHE-Delft P.O. Box 3015 2610 DA, Delft, The Netherlands; 3 Harbour, Coastal and Offshore Engineering, Deltares, PO Box 177, 2600 MH Delft, The Netherlands; 4 Water Engineering and Management, Faculty of Engineering Technology, University of Twente, PO Box 217, 7500 AE Enschede, The Netherlands

**Keywords:** Physical oceanography, Civil engineering

## Abstract

The development of effective coastal adaptation strategies and protection schemes is a major challenge for coastal zone managers and engineers, not only because the coastal zone is the most populated and developed land zone in the world, but also due to projected climate change impacts. A priori knowledge of the so called depth of closure (DoC) is, more often than not, a pre-requisite to understand and model coastal morphological response to wave forcing, which in turn enables the design of appropriate coastal adaption/protection measures. In the absence of long term measurements of coastal profile data, the DoC is often computed using Hallermeier’s formulations or derivatives thereof, for applications around the world. However, there are two major unresolved issues associated with computing the DoC in this way: the accuracy of the wave data required for reliable DoC computations, and the generic applicability of the coefficients used in DoC equations. This study exploits the availability of DoCs derived from multiple measurements of coastal profiles and wave data along the Japanese coast together with wave reanalysis products to evaluate the validity of DoC calculation approaches. Results show that the accuracy of computed DoC values determined using wave reanalysis data is limited, particularly when the spatial resolution of the wave reanalysis data is lower. Furthermore, coefficients of DoC equations proposed in previous and present studies appear to be location specific and points toward the need for a concerted worldwide meta-analysis that compares observed and derived DoC in order to derive a globally applicable formulation for DoC computations.

## Introduction

The coastal zone is the most heavily populated land zone in the world^1–3^ with about 10% of the global population living within the low elevation coastal zones of the world^4^. As a direct result, there is vast amount of developments and infrastructure located within the coastal zone. While these coastal communities, developments and infrastructure are already threatened with coastal hazards like severe storms, king tides, and storm surges, climate change driven variations in mean seal level (i.e. sea level rise), waves and surges are expected to result in more frequent and more severe coastal flooding and erosion^5,6^. Therefore, the development of appropriate coastal zone management and protection strategies will be even more important in the future than it already is at present. However, the lack of phenomenological understanding and accurate prediction methods of long-term (i.e. decades or longer) coastal morphological response to forcing is a major challenge for sustainable coastal zone management.

The development of coastal adaptation strategies (e.g. establishment of setback lines) or the design of coastal settlement/infrastructure protection schemes (e.g. hard engineering structures) is often done via the application of numerical modelling approaches. These modelling approaches can vary from simple analytical expressions (e.g. the Bruun Rule^7^), to one-line models (e.g. GENESIS^8,9^, UNIBEST^9^), to semi-empirical models (e.g. SBEACH^10^), and to very sophisticated, three-dimensional coastal morphodynamic models (e.g. Delft3D^11^, MIKE21^12^, XBeach^13^). One of the key parameters used in many of these models (except in the case of fully process based models) is the depth of closure (DoC, *h*_*c*_)^6–9,14–18^. The DoC, which increases with the time scale under consideration^19^, is defined as the seaward depth at which sediment transport and consequent bed level change are insignificant. The time scale dependency of the DoC is illustrated by Cowell and Kinsela^20^ who developed a framework for investigating shoreface morphologic-response timescales based on Hallermeier^21,22^ and Stive and deVriend^23^ as follows:

- The upper shoreface, which is affected by wave breaking and surf zone processes;
- The active shoreface, which implicitly includes the upper shoreface, and is characterized as having statistically stationary geometry at the observation timescale; and
- The lower shoreface, which is affected by wave shoaling, where profile response is immeasurable or insignificant at the observation timescale.

The timescales of upper, active, and lower shoreface responses could be determined as less than years, years to decades, and decades to centuries, respectively. In this framework, the DoC formulation by Hallermeier^21,22^ would typically be located around the seaward limit of the upper shoreface. While many studies have focused on DoC^21,22,24–30^, the DoC is still often computed using Hallermeier’s^21,22^ formulations or derivatives thereof, which were intended to capture the limit of profile variability at annual timescales.

Hallermeier^21^ used the Shields parameter to derive the following equation regarding *h*_*c*_:

1 $$k{h}_{c}{\sinh }^{2}(k{h}_{c}){\tanh }^{2}(k{h}_{c})(1+\frac{(2k{h}_{c})}{\sinh (2k{h}_{c})})=\frac{329\rho {H}_{0}^{2}}{({\rho }_{s}-\rho ){L}_{0}^{2}},$$

where *k* is the wave number; *H*_0_ and *L*_0_ are the deep water wave height and length, respectively; and *ρ* and *ρ*_s_ are the densities of fluids (1.03 kg m^−3^, salt water) and sand particles (2.65 kg m^−3^), respectively. The value of *kh*_*c*_ can be obtained numerically if deep water wave values are given; then *h*_*c*_ can be solved using the dispersion relationship^31^. Hallermeier^21^ found that *h*_*c*_ obtained from Eq. (1) agreed well with laboratory measurements. Following a detailed analysis, Hallermeier^21^ proposed the well-known equation for *h*_*c*_ as:

2 $${h}_{c}=a{H}_{e}+b({{H}_{e}}^{2}/g{{T}_{e}}^{2})$$

where *H*_*e*_ is the nearshore significant wave height that is exceeded for only 12 hours a year, *T*_*e*_ is the associated wave period, and *g* is the gravitational acceleration. Coefficients *a* and *b* are given by 2.28 and −68.5, respectively (hereon, these coefficient values are referred to as “HM”). Birkemeier^24^ applied Eq. (2) to beach-nearshore profile data and wave data at a depth of 18 m from June 1981 to December 1982 also at the USACE Field Research Facility, Duck, North Carolina (USA) and determined the coefficients *a* and *b* to be 1.75 and −57.9 (hereon, these values are referred to as “BM1”). Birkemeier^24^ noted that a reasonable fit could also be obtained when using *H*_*e*_ alone but by forcing the regression through the origin. In this latter approach, the values of coefficients determined for *a* and *b* are 1.57 and 0.0 (referred to as “BM2”). Nicholls *et al*^25^. also evaluated the applicability of Eq. (2) over periods up to four years using 12 years (July 1981 to July 1993) of beach profile data collected at Duck using a time-dependent form of Eq. (2):

3 $${h}_{c,t}=a{H}_{e,t}+b({{H}_{e,t}}^{2}/g{{T}_{e,t}}^{2})$$

where *H*_*e,t*_ is the significant wave height that is exceeded for only 12 hours in *t* years and *T*_*e,t*_ is the associated wave period (hereinafter, time-dependent *h*_*c,t*_, *H*_*e,t*_ and *T*_*e,t*_ are expressed as *h*_*c*_, *H*_*e*_ and *T*_*e*_ because we conducted only time-dependent analysis). Nicholls *et al*^25^. showed that the observed DoC was 69% of the estimated DoC when using Eq. (3) with Hallermeier’s^21,22^ coefficients; however, they indicated that Hallermeier’s^21,22^ approach provides a reasonable representation of DoC for periods up to four years. They also demonstrated that the DoC increases with observation time scale.

Most DoC studies have focused only on a single transect profile because repeated profile data are not generally available. An exception is the recent study reported by Patterson and Nielsen^32^, which used multiple-transect profile data from 1966 to 2012 and wave data measurements since 1986 at northern Gold Coast, Australia. Also, Hartman and Kennedy^27^ investigated DoC for a 2 km long beach region using the Joint Airborne Lidar Bathymetry Technical Center of Expertise (JALBTCX) dataset collected for sandy coastlines in Florida over the past decade. They used a bathymetry dataset containing 19 surveys from 2004–2012; however, their analysis was limited by wave data sourced from Wave Information Studies (WIS) wave hindcast models which did not include the effects of tide and storm surge. They concluded that the accuracy of DoC calculations could be further improved using wave hindcast models that contain storm surge water level variations as well as those with higher nearshore resolution^27^. Valiente *et al*^29^. compared the DoC observed using beach profile data at Perranporth, UK, to that estimated using Eq. (3) fed by the output of a 8-km resolution regional wave forecast model based upon WAVEWATCH III from 2010–2016, and concluded that DoC was estimated with adequate accuracy by Eq. (3). Ideally the DoC at a given location would be estimated using repeated profile measurements over a long period of time (decade or more). However, such data is hardly available, and therefore, while the applicability of Eq. (3) appears to have been validated at a few sandy coasts, its validity in other parts of the world, and especially over longer time scales still remains unknown.

**Figure 1:**
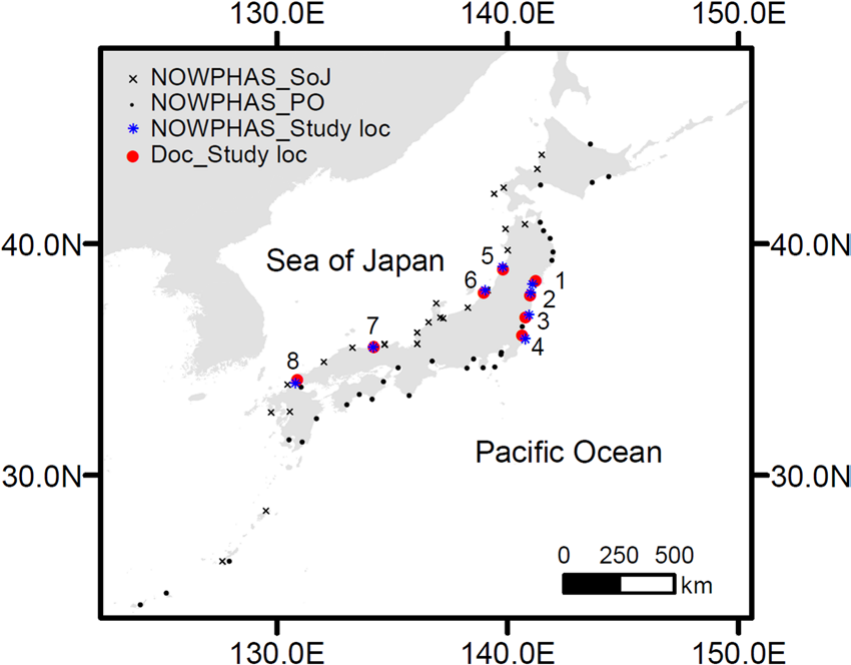
Locations of wave observation (NOWPHAS) stations used for the wave analysis, and the eight beach profile measurement sites together with the closest NOWPHAS stations used in the DoC analysis. Details on study sites are given in Table 1.

The DoC equations formulated by Hallermeier’s^21,22^ approach are widely used; nevertheless, there are still two major unresolved issues associated with computing the DoC in this way: (i) the accuracy of the wave data required for reliable DoC computations, and (ii) the generic applicability of the coefficients used in DoC equations. Here we assess the applicability of Eq. (3) by comparing the DoCs measured at eight study locations around Japan (Table 1 and Fig. 1) with those computed using the equation with both observed and reanalysis wave data, to gain new insights on these knowledge gaps. First we evaluate the reanalysis wave data (i.e., Coastal Wave Model, CWM^33^; WAVEWATCH III^34^; and ERA5^35^) by comparing them with the observed wave data (i.e., the Nationwide Ocean Wave information network for Ports and HArbourS, NOWPHAS^36^) and then use both the reanalysis and observed wave data to estimate DoCs. Finally the applicability of the DoC equation is evaluated by comparing computed DoCs with Uda’s^30^ and others’ unique years-to-decadal measured DoCs around the Japanese coast (see Fig. S1).

**Table 1:** Details of beach profile and wave observation data for the eight locations shown in Fig. 1. See also Fig. S1 for an example of profile data.

Location No.	1	2	3	4	5	6	7	8
Location name	Sendai	Soma	Onahama	Kashima	Sakata	Niigata	Tottori	Ainoshima
Longitude [deg]	141.2190	140.9852	140.7731	140.6290	139.8047	138.9640	134.2054	130.8920
Latitude [deg]	38.3971	37.7856	36.8219	36.0501	38.9102	37.8896	35.5417	34.1363
Profile data period [year]	1973–1984 (11 years)	1977–1991 (14 years)	1979–1988 (9 years)	1983–1992 (9 years)	1975–1990 (15 years)	1982–1989 (7 years)	1974–1985 (11 years)	1980–1987 (7 years)
Profile measurement timing	2	3	2	10	15	3	10	6
Number of profile transects	5	2	1	Bathymetry	2	4	Bathymetry (140)	1
Observed DoC [m]	9	10	9	8	> 15	12	14	7
NOWPHAS station code	205	214	206	207	102	110	304	406
NOWPHAS available data period [year]	1979-present	1982- present	1980-present	1972-present	1970-present	1982- present	1979-present	1975-present
He12 [m]	4.99	5.01	5.85	5.97	7.56	5.36	5.79	4.20
Te12 [s]	13.3	11.2	13.8	12.3	10.4	9.2	9.4	10.3
Water depth of NOWPHAS measurement [m]	20.0	16.0	20.0	24.5	20.4	22.7	30.0	20.7
Distance between Study sites and NOWPHAS [km]	21	9	17	20	11	11	7	17

## Results and Discussion

### Evaluation of reanalysis wave data

Spatial distributions of reanalysis wave heights (*H*_*mean*_ and *H*_*e*_) and periods (*T*_*mean*_ and *T*_*e*_) for reanalysis wave datasets (CWM^33^, WAVEWATCH III^34^, and ERA5^35^) around Japan are shown in Figs. S2 and S3, respectively. The CWM dataset is computed only around Japan with higher resolution of 5 to 10 km, and WAVEWATCH III and ERA5 are often-used and computed globally with relatively lower resolution (Table S1). It is apparent that mean and 12 hour exceedance wave distributions are larger in the Pacific Ocean and smaller in the Japan Sea. The wave height distributions show approximately similar distributions for all models; however, the ERA5 derived wave periods are smaller compared to those of CWM and WAVEWATCH III.

**Figure 2:**
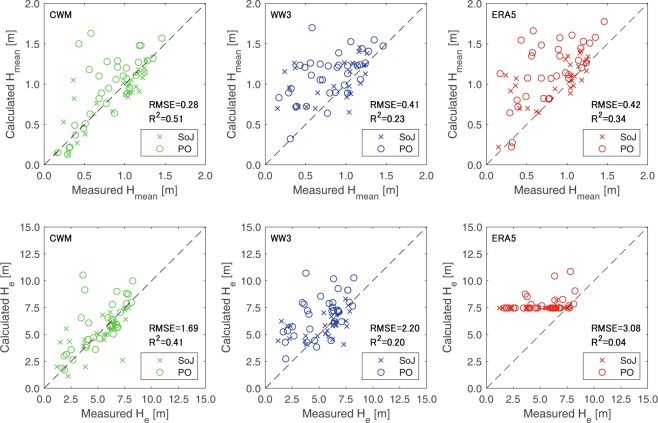
Comparisons between observed Nationwide Ocean Wave information network for Ports and HArbourS (NOWPHAS) and reanalysis wave height data (CWM, WAVEWATCH III, and ERA5) in the period for 2005–2009. SoJ and PO indicate wave data obtained in the Sea of Japan and the Pacific Ocean sides, respectively.

**Figure 3:**
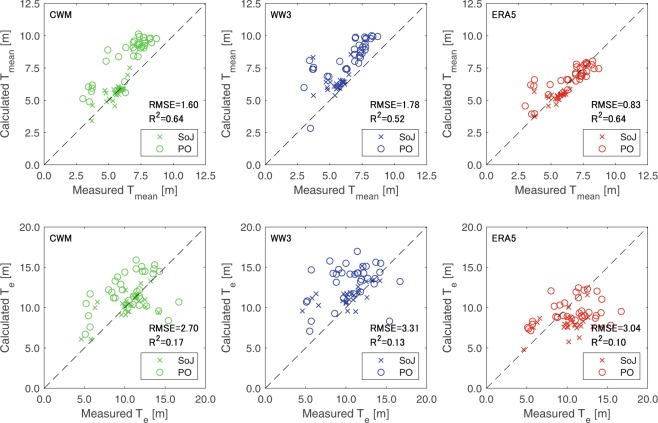
Same as Fig. 2 but for wave period data.

Reanalysis values during the period of 2005 to 2009 are also compared with NOWPHAS observation data at 62 measurement points along the coast (see Fig. 1) as shown in Figs. 2 and 3. The period bounded by 2005 and 2009 was selected for this analysis due to two reasons: (i) before the mid 2000s and after the 2011 Tohoku Tsunami, the number of the wave stations and the data acquisition rate of NOWPHAS network was sub-optimal, and (ii) the WAVEWATCH III reanalysis extends only up to 2009. Overall, CWM gives a good agreement with the NOWPHAS data. ERA5 shows a better agreement than WAVEWATCH III for *H*_*mean*_ and *T*_*mean*_ obtained from NOWPHAS data; however, *H*_*e*_ and *T*_*e*_ obtained from both global reanalysis products do not agree well with that computed from NOWPHAS data. The better agreement of CWM appears to be linked to the spatial resolution of the reanalysis data because most of the model/data disparities occur in small wave shelter zones such as bays. Such locations are therefore not included in the study sites in the DoC analysis.

### DoC evaluation

**Figure 4:**
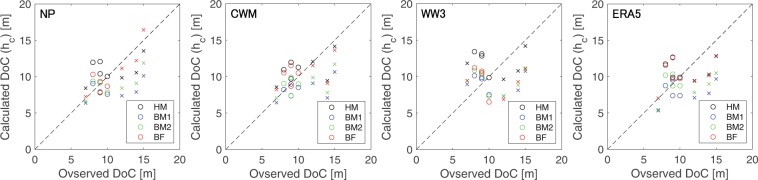
Comparisons of observed DoCs and DoCs derived from Eq. (3) with *H*_*e*_ of observed (NOWPHAS) and reanalysis wave height data (CWM, WAVEWATCH III, and ERA5) at the eight study locations. Black, blue, green, and red symbols indicate plots for HM, BM1, BM2, and BF models, and circle and cross symbols indicate plots for the Sea of Japan and the Pacific Ocean side locations, respectively. The observed DoC values are also shown in Table 1.

**Figure 5:**
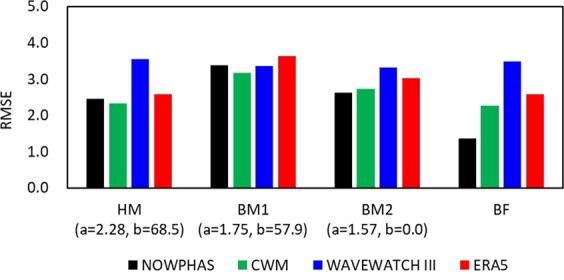
Root mean square errors (RMSE) of DoCs derived from Eq. (3). Note that the data period of CWM is different to that of the period of profile measurement due to data availability. See also Fig. 4.

The DoC (*h*_*c*_) were estimated using the 12-hour exceedance wave data during the same period as the profile measurements for determining the observed DoC (see Table 1 and Fig. S1). Similar to previous studies^25,27,29^, DoC estimates obtained using Hallermeier^21,22^ appear to be acceptable (Fig. 4). It is noteworthy that all three DoC models (HM, BM1, and BM2) tend to overestimate DoC along the Pacific Ocean side and underestimate DoC along the Sea of Japan side. Our results also show that the DoC RMSEs, except for WAVEWATCH III, are smaller for HM compared to BM1 and BM2 (Fig. 5). Among the DoCs calculated using wave reanalysis data, those obtained from high resolution CWM data consistently outperforms the coarser resolution ERA5 and WAVEWATCH III data, implying that wave model resolution does matter for DoC calculations. Furthermore, the coefficients *a* and *b* of the best fit model for the DoCs calculated using observed wave data are 1.14 and 144.8, respectively, while those for the DoCs calculated using reanalysis wave data display wide ranges of 1.20–2.32 and −77.7–85.1, respectively (hereon, the best-fit coefficients are referred to as “BF”) as shown in Table S2, indicating that coefficients proposed by previous studies are location dependent as Birkemeier^24^ also mentioned. Among the BF DoCs calculated using wave reanalysis data, the DoC calculated using CWM has the smallest RMSE, while the WAVEWATCH III DoC has the largest RMSE, reinforcing the superior performance of the high resolution CWM data compared to the lower resolution global reanalysis products in terms of DoC calculations. Note that the CWM data period 2005–2009 shown in Figs. S2 and S3 was used for the estimation since CWM results were not available for the profile measurement period.

We found that the coefficients *a* and *b* of HM, BM1, and BM2 and the four BFs for the wave data display a positive linear relationship (*R*^2^ = 0.87, *p* < 0.005), although the number of their data points is limited to seven (see Table S2). We also found that the Hallermeier’s coefficients for 19 datasets classified according to parameters including bed slope, sediment size, and wave geometry^21^ also display a positive linear relationship, which suggests that DoC may be better estimated by determining suitable coefficients using such aggregated parameters.

## Conclusions

In this study we combined DoC and continuous wave observations from multiple sandy beaches in Japan with wave reanalysis data to evaluate the relative merits of commonly used empirical equations for determining DoCs. Results indicated that the accuracy of DoCs determined using reanalysis wave data is limited by the spatial resolution of the reanalysis. Based on our results, we suggest that DoC equation coefficients proposed in previous and present studies may in fact be location dependent, and hence, not generically applicable to coastal regions globally, although it should be noted that the low number of study sites (8) in this study does limit our ability to draw more definitive conclusions. The outcomes of this study point towards the need for a worldwide meta-analysis that compares observed and derived DoC in order to derive a globally applicable formulation for DoC computations.

## Methods

### Wave analysis

Two-hourly time-series of significant wave height and period data from the Nationwide Ocean Wave information network for Ports and HArbourS (NOWPHAS)^36^, provided by the Ports and Harbours Bureau, Ministry of Land, Infrastructure, Transport, and Tourism (MLIT), were used in this study. Five NOWPHAS stations were in operation in 1970 and this increased to more than sixty stations by the mid 2000s (Fig. 1). In the latter half of the 2000 decade, the temporal data acquisition rate was more than 80% at most locations. However, following the 2011 Tohoku Tsunami, data was not recorded for several years in tsunami affected areas. NOWPHAS wave data is collected by ultrasonic wave gauges, step-type wave gauges, or Doppler-type directional wave meters. Available wave data period, water depth of wave measurements, and wave measurement instrument type at the study sites are shown in Table 1. We note that the available wave data period does not cover the entire profile measurement period for several study sites.

Reanalysis wave datasets, i.e. significant height and mean wave period, used in this study included the Coastal Wave Model (CWM) data from the Japan Meteorological Agency (JMA)^33^, WAVEWATCH III data from the National Oceanic and Atmospheric Administration (NOAA)^34^, and ERA5 data from the European Centre for Medium-Range Weather Forecasts (ECMWF)^35^ (Table S1). The CWM dataset was generated using the third generation wave model MRI-III (since March 2002) and wind grid point values forecast by the JMA operational weather model, upgraded in May 2007^33,37^. The CWM has hindcast real-time wave conditions around Japan using reanalysis wind data twelve hourly from March 2002 to May 2007 and six hourly since May 2007. An 84 hour (three hourly) wave forecast is also provided. The spatial resolution of the model also increased from 0.1 to 0.05° in May 2007 and is high as compared to other reanalysis data. WAVEWATCH III provides three hourly data from 1979–2009 at a spatial resolution of 0.5° and ERA5 provides hourly data at 0.5° from 1979 to the present. Mean and 12-hour exceedance significant wave heights and periods from 2005–2009 (i.e., *H*_*mean*_, *H*_*e*_, *T*_*mean*_, and *T*_*e*_, respectively) were calculated for the observed and reanalysis wave data at 62 measurement points with a temporal acquisition rate exceeding 80%. The reanalysis wave data were compared with observations.

### DoC analysis

More than 50 Japanese beaches for which DoCs were presented in Uda^30^ were initially considered as potential study sites for this study. The profiles had been generally measured by level surveys in the intertidal area and by acoustic bathymetric survey in the subtidal area^30^. To improve the accuracy of our analysis, this data set was first narrowed down by considering only the sites for which (i) beach profile data for at least a five-year period, and (ii) wave data within 20 km of profile measurement locations were available. Concerning the accuracy of DoC observations, Valiente *et al*^29^. indicated five criteria of which the most used criteria is the minimum depth of the uncertainty limit for detecting significant morphologic change (e.g., Δ*d* < 0.03 m^24^ and 0.06 m^25^ for the data obtained by Coastal Research Amphibious Buggy (CRAB) and Δ*d* < 0.14 m^29^ for the data obtained by single-beam echo-sounder). Uda^38^ demonstrated that the accuracy of annual profile data measured by acoustic surveys from 1976–1987 along the coastline of the Niigata Prefecture was 0.28 m; and therefore, the observed DoCs used herein can be considered to have an accuracy of 0.28 m.

Based on wave data and profile data availability, eight study locations were ultimately chosen for this study (see Table 1 and Fig. 1). At the selected sites, DoC was calculated using Eq. (3) with both observed and reanalysis wave data, and then the derived DoC was compared with observed DoC. The best-fit model of Eq. (3) for each reanalysis dataset was obtained by determining the coefficients *a* and *b* using minimum RMSE (see Table S2).

## Supplementary information

Supplementary information.

Dataset 1.

## Data Availability

Data used to derive the tables and figures shown in this manuscript is available in xlsx format. Original wave reanalysis data is available from CWM, WAVEWATCH III, and ERA5.
