# Supplementary material for: An assessment of measured and computed depth of closure around Japan
Source: Sci Rep. 2020 Feb 19;10:2987. doi: 10.1038/s41598-020-59718-5 (PMC7031332; doi:10.1038/s41598-020-59718-5)
Supplement: Supplementary file 1 — Supplementary information. [file 41598_2020_59718_MOESM1_ESM.docx]

An assessment of measured and computed depth of closure around Japan

Keiko Udo^1*^, Roshanka Ranasinghe^2,3,4^, and Yuriko Takeda^1^

^1^ *International Research Institute of Disaster Science, Tohoku University, 468-1 Aoba, Sendai 980-8572, Japan.*

^2^ *Department of Water Science and Engineering, IHE-Delft P.O. Box 3015 2610 DA Delft, The Netherlands.*

^3^ *Harbour. Coastal and Offshore Engineering, Deltares, PO Box 177, 2600 MH Delft, The Netherlands*.

^4^ *Water Engineering and Management, Faculty of Engineering Technology, University of Twente, PO Box 217, 7500 AE Enschede, The Netherlands*.

***** udo@irides.tohoku.ac.jp

**Supplementary Information**

**Tables**

**Table S1.** Details of observed and reanalyzed wave data used in the study.

| Dataset (Model) | Type of data | Temporal resolution | Spatial resolution | Period |
| --- | --- | --- | --- | --- |
| NOWPHAS | Observed | 2 hourly | 62 points as of 2005 along coasts in Japan (see Figure 2) | 1970-present |
| Coastal Wave Model (CWM) | Reanalyzed | 12 hourly (Mar 2002-May 2007), 6 hourly (May 2007-) | 0.1 degree from Mar 2002 to May 2007 and 0.05 degree from June 2007 around Japan (20-50N, 120-150E) | 2002-present |
| WAVEWATCH III | Reanalyzed (Phase 2) | 3 hourly | 0.5 degree, Global | 1979-2009 |
| ERA5 | Reanalyzed | Hourly | 0.5 degree, Global | 1979-present |

**Table S2.** Coefficients *a* and *b* of HM, BM1, and BM2 and four BF models.

| Model | *a* | *b* |
| --- | --- | --- |
| HM | 2.28 | -68.5 |
| BM1 | 1.75 | -57.9 |
| BM2 | 1.57 | 0.0 |
| BF_NOWPHAS | 1.14 | 144.8 |
| BF_CWM | 1.87 | -7.3 |
| BF_WAVEWATCH III | 1.20 | 85.1 |
| BF_ERA5 | 2.32 | -77.7 |

**Figures**


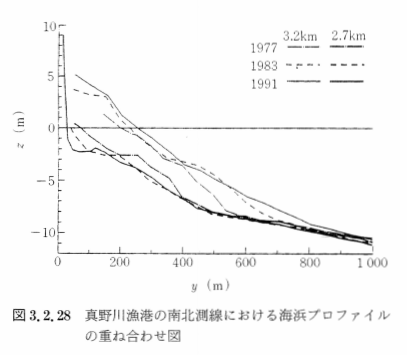
**
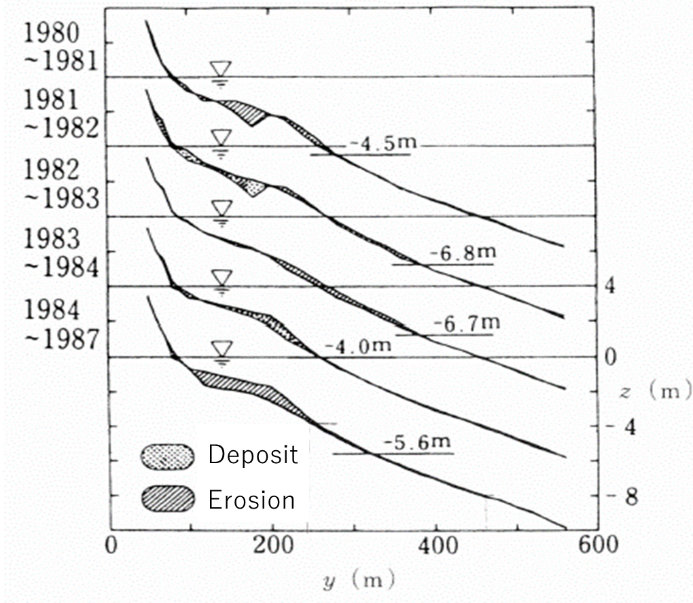
**

**Figure S1.** Examples of beach profile figures^27^ used to determine the depth of closure (DoC) at Soma (left, location No. 2) and Ainoshima (right, location No. 8; revised from Uda^27^). See Table 1 and Fig. 1 for the study site details and locations.


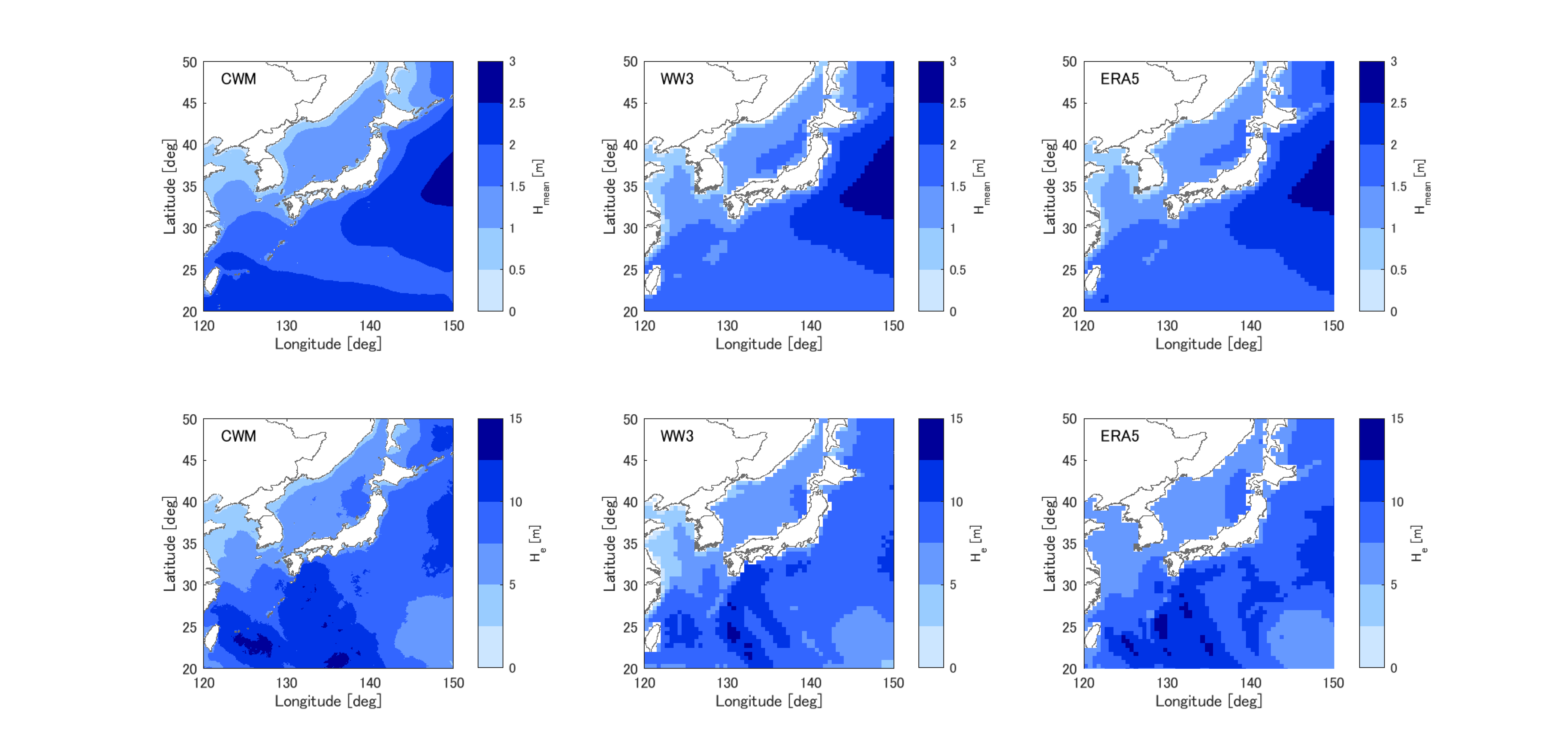


**Figure S2.** Spatial distribution of mean and 12 hour exceedance wave height (*H_mean_* and *H_e_*, respectively) from reanalysis wave data (CWM, WAVEWATCH III, and ERA5) during 2005–2009.


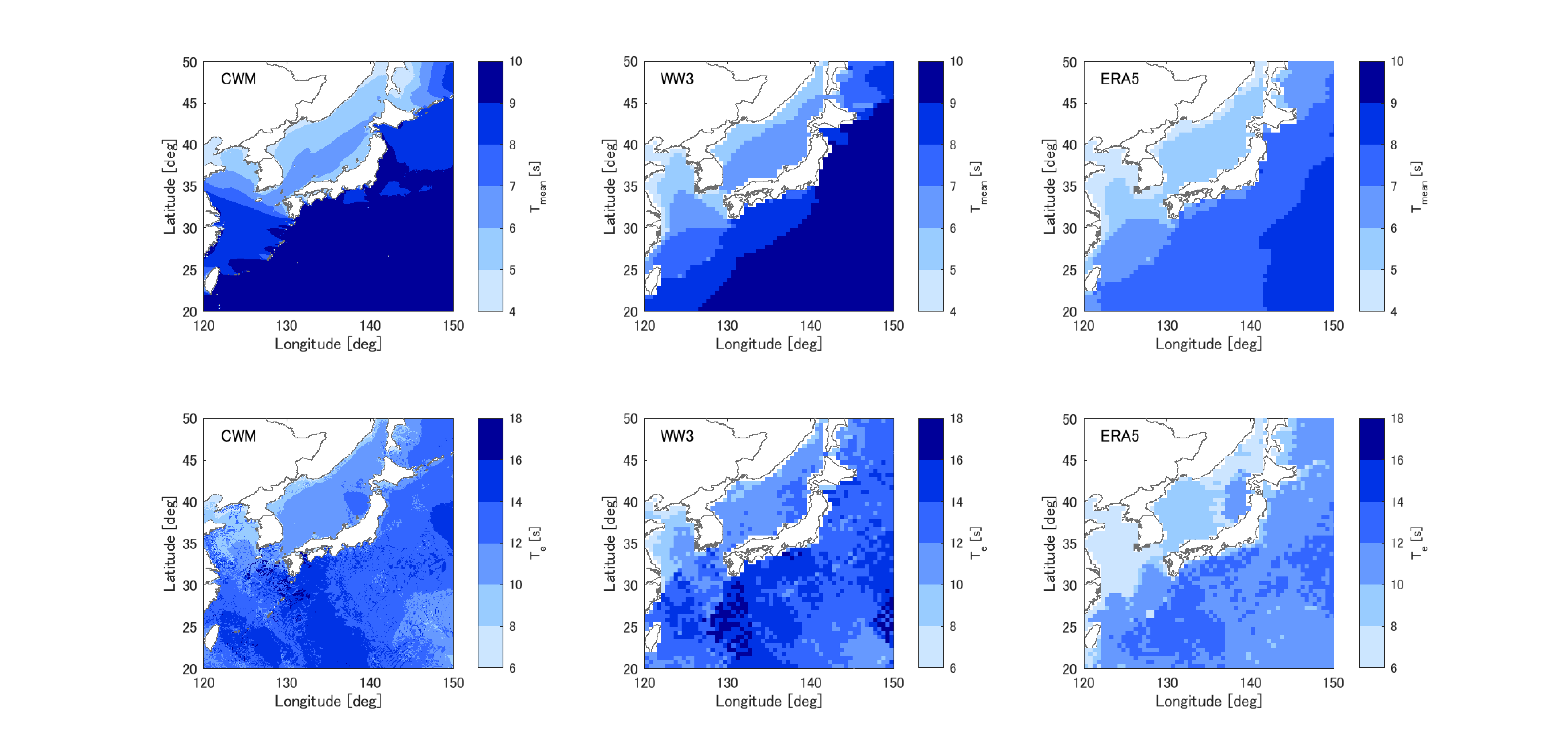


**Figure S3.** Same as Fig. S2 but for wave period data.
